# Supplementary material for: Distinct functions of microtubules and actin filaments in the transportation of the male germ unit in pollen
Source: Nat Commun. 2024 Jun 27;15:5448. doi: 10.1038/s41467-024-49323-9 (PMC11211427; doi:10.1038/s41467-024-49323-9)
Supplement: Supplementary file 1 — Supplementary Information [file 41467_2024_49323_MOESM1_ESM.pdf]

Supplementary Information

Distinct functions of microtubules and actin filaments in the transportation of MGU in pollen

Xiangfei Wang[1,3], Tonghui Li[1,3], Jiuting Xu[1,3], Fanfan Zhang[1], Lifang Liu[1], Ting Wang[1], Chun Wang[1], Haiyun Ren[1,2*] & Yi Zhang[1*]

[1]Key Laboratory of Cell Proliferation and Regulation Biology of Ministry of Education, College of Life Sciences, Beijing Normal University, 100875 Beijing, China.

[2]Center for Biological Science and Technology, Guangdong Zhuhai-Macao Joint Biotech Laboratory, Beijing Normal University, 519087 Zhuhai, China.

[3]These authors contributed equally

[*]Corresponding author

Haiyun Ren (hren@bnu.edu.cn) and Yi Zhang (yi.zhang@bnu.edu.cn)

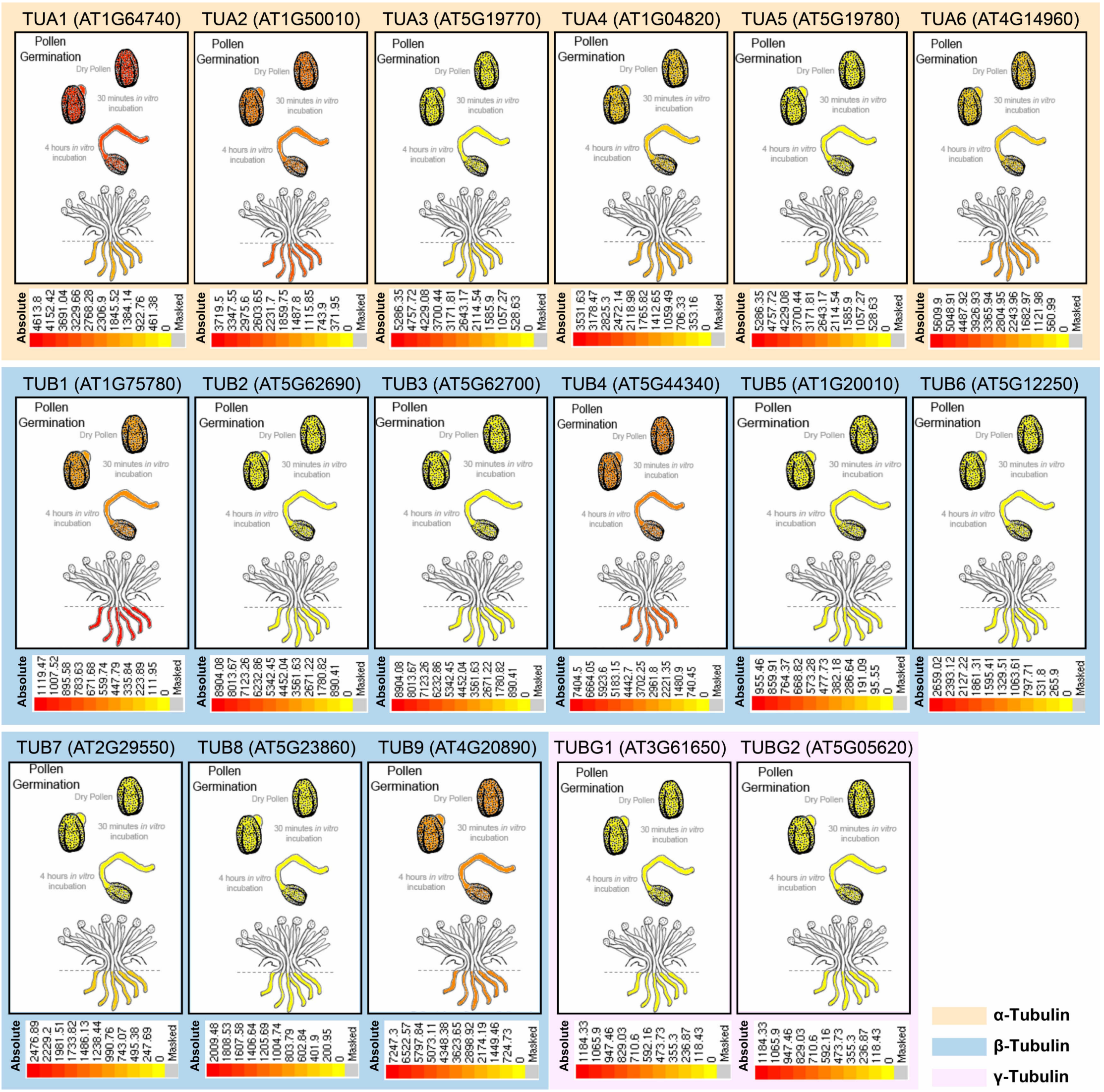

Supplementary Figure 1. The expression levels of α, β, and γ-tubulins in Arabidopsis pollen grains and tubes.

TUA1, *TUB1*, *TUB4* and *TUB9* show high expression in pollen. Expression levels of Tubulin genes in pollen and pollen tubes were determined from the e-FP browser (https://bar.utoronto.ca/efp//cgi-bin/efpWeb.cgi).

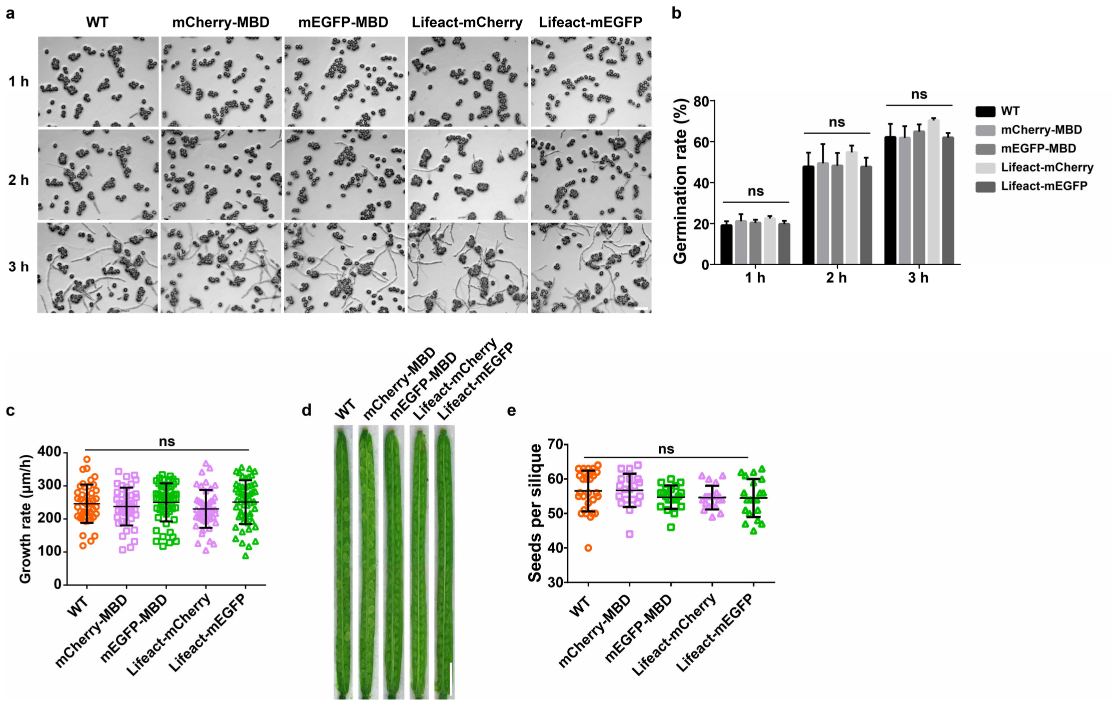

Supplementary Figure 2. The mCherry-MBD, mEGFP-MBD, Lifeact-mCherry and Lifeact-mEGFP lines have minor impact on pollen germination, pollen tube elongation and fertility.

a Representative images of wild type and mCherry-MBD, mEGFP-MBD, Lifeact-mCherry and Lifeact-mEGFP-expressing pollens cultured on germination medium for 1, 2 or 3 h. Bar, 100 μm. **b** Quantification of the germination rate of wild type and the mCherry-MBD, mEGFP-MBD, Lifeact-mCherry and Lifeact-mEGFP lines. The experiments were repeated for three times with more than 800 pollen grains per replicate. ns, not significant; two-tailed Student's *t* test. **c** Quantification of pollen tube growth rate as cultured in (**a**). WT (n = 43), mCherry-MBD (n = 42), mEGFP-MBD (n = 54), Lifeact-mCherry (n = 48) and Lifeact-mEGFP (n = 53). **d, e** Number of seeds per silique compared between different marker lines. n = 28 siliques from WT, n = 20 siliques from mCherry-MBD, mEGFP-MBD, Lifeact-mCherry and Lifeact-mEGFP lines. Bar = 2 mm. Values are mean ± SD; ns, not significant; two-tailed Student's *t* test in (**b**), (**c**) and (**e**). Source data are provided as a Source Data file.

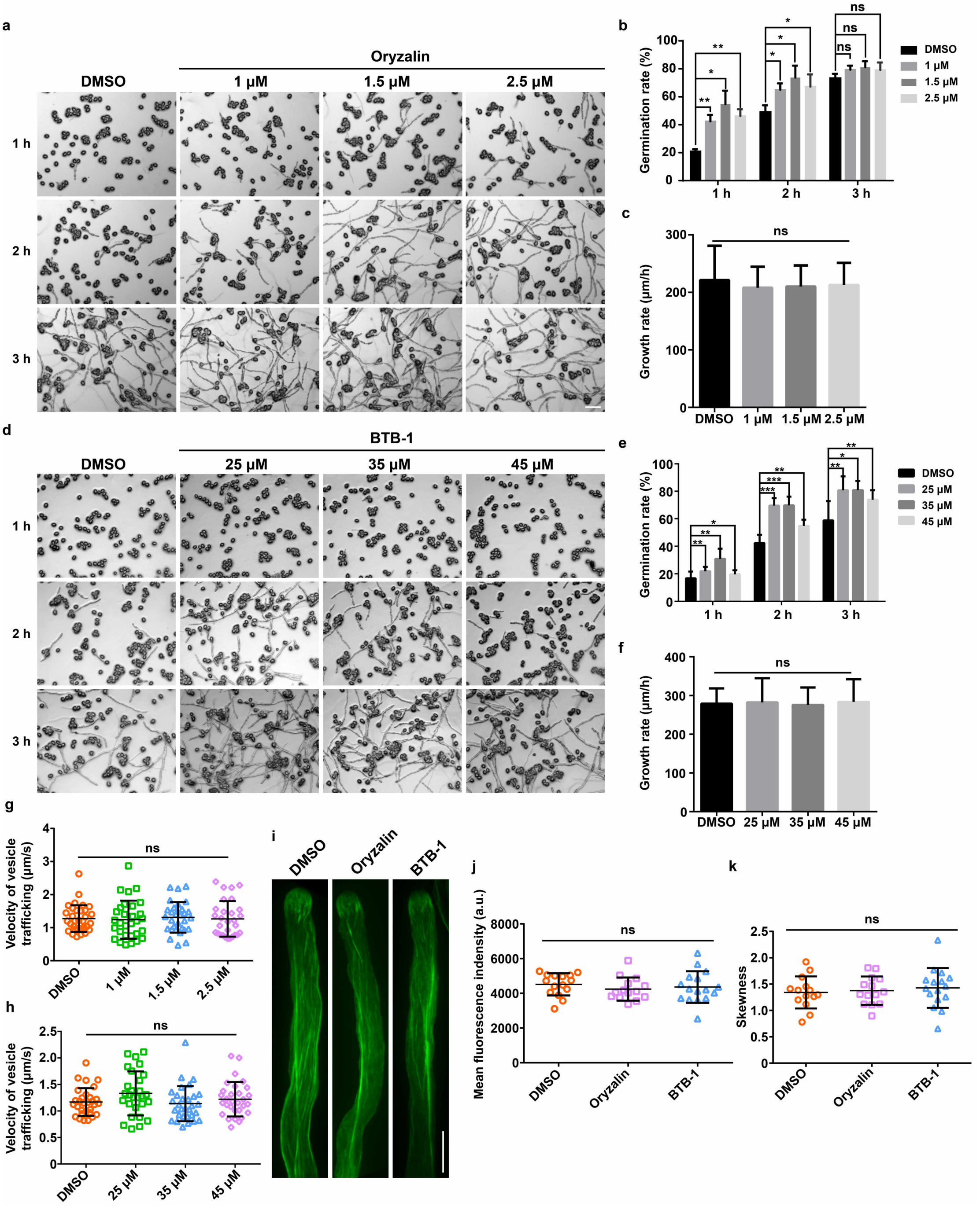

Supplementary Figure 3. The effects of oryzalin or BTB-1 treatment on pollen germination rate, the growth rate of pollen tubes, cytoplasmic streaming and actin filament organizations.

a Representative images of wild-type pollen cultured on media supplemented with DMSO or oryzalin (1.0-2.5 μM) for 1-3 h. Bar, 100 μm. **b** Quantification of pollen germination rate as cultured in (**a**). The experiments were repeated for three times with more than 800 pollen grains per replicate. **c** Quantification of pollen tube growth rate as cultured in (**a**). n = 100 pollen tubes for each treatment. **d** Representative images of wild-type pollen cultured on media supplemented with DMSO or BTB-1 (25-45 μM) for 1-3 h. Bar, 100 μm. **e** Quantification of pollen germination rate as cultured in (**d**). The experiments were

repeated for three times with more than 800 pollen grains per replicate. **f** Quantification of pollen tube growth rate as cultured in (**d**). DMSO (n = 51), 25 μm BTB-1 (n = 76), 35 μm BTB-1 (n = 92), 45 μm BTB-1 (n = 87). **g** Quantification of the velocity of vesicle trafficking in DMSO and oryzalin-treated pollen tubes. DMSO (n = 33), each oryzalin-treatment (n = 30). **h** Quantification of the velocity of vesicle trafficking in DMSO and BTB-1-treated pollen tubes. DMSO (n = 30), 25 μm BTB-1 (n = 28), 35 μm BTB-1 (n = 30), 45 μm BTB-1 (n = 30). **i** Representative images of Lifeact-mEGFP pollen cultured on media supplemented with DMSO, 1.5 μM oryzalin or 35 μM BTB-1 for 3 h. Bar, 10 μm. **j** Mean fluorescence indensity of actin filaments in DMSO-, oryzalin- or BTB-1-treated pollen tubes. DMSO (n = 15), Oryzalin (n = 14), BTB-1 (n = 16). **k** Skewness of actin filaments in DMSO-, oryzalin- or BTB-1-treated pollen tubes. DMSO (n = 14), Oryzalin (n = 14), BTB-1 (n = 16). Values are mean ± SD; * $P < 0.05$, ** $P < 0.01$, *** $P < 0.001$, ns, not significant; two-tailed Student's *t* test in (**b**), (**c**), (**e-h**), (**j**), (**k**). Source data are provided as a Source Data file.

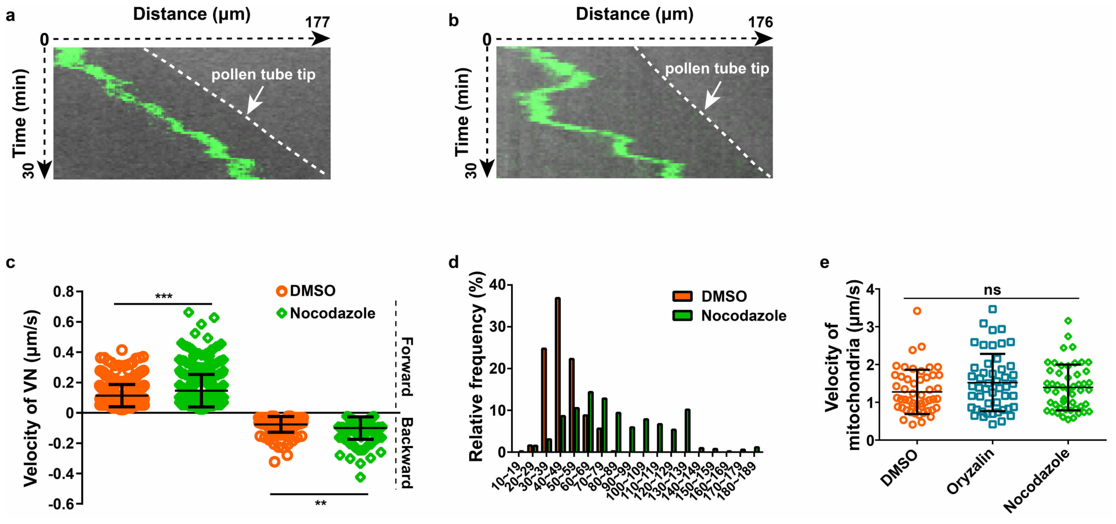

Supplementary Figure 4. The effects of nocodazole treatment on the movement of VN in pollen tubes.

a, b Kymograph analysis of the signals of VN in DMSO (**a**) or nocodazole-treated growing pollen tubes (**b**). The pollen tube tip is indicated with an arrow. **c** Quantification of VN's movement velocity in DMSO and nocodazole-treated pollen tubes. Velocity of VN forward movement, DMSO (n = 470), Nocodazole (n = 497); velocity of VN backward movement, DMSO (n = 157), Nocodazole (n = 152), n from 10 cells for each treatment. **d** The frequency distribution of the distance of VN to the tip of DMSO and nocodazole-treated pollen tubes. **e** Quantification of the velocity of mitochondria movement in DMSO, oryzalin or nocodazole-treated pollen tubes. DMSO (n = 50), Oryzalin (n = 49), Nocodazole (n = 50) from 10 cells for each treatment. Values are mean ± SD; ** $P < 0.01$, *** $P < 0.001$, ns, not significant; two-tailed Student's t test in (**c**) and (**e**). Source data are provided as a Source Data file.

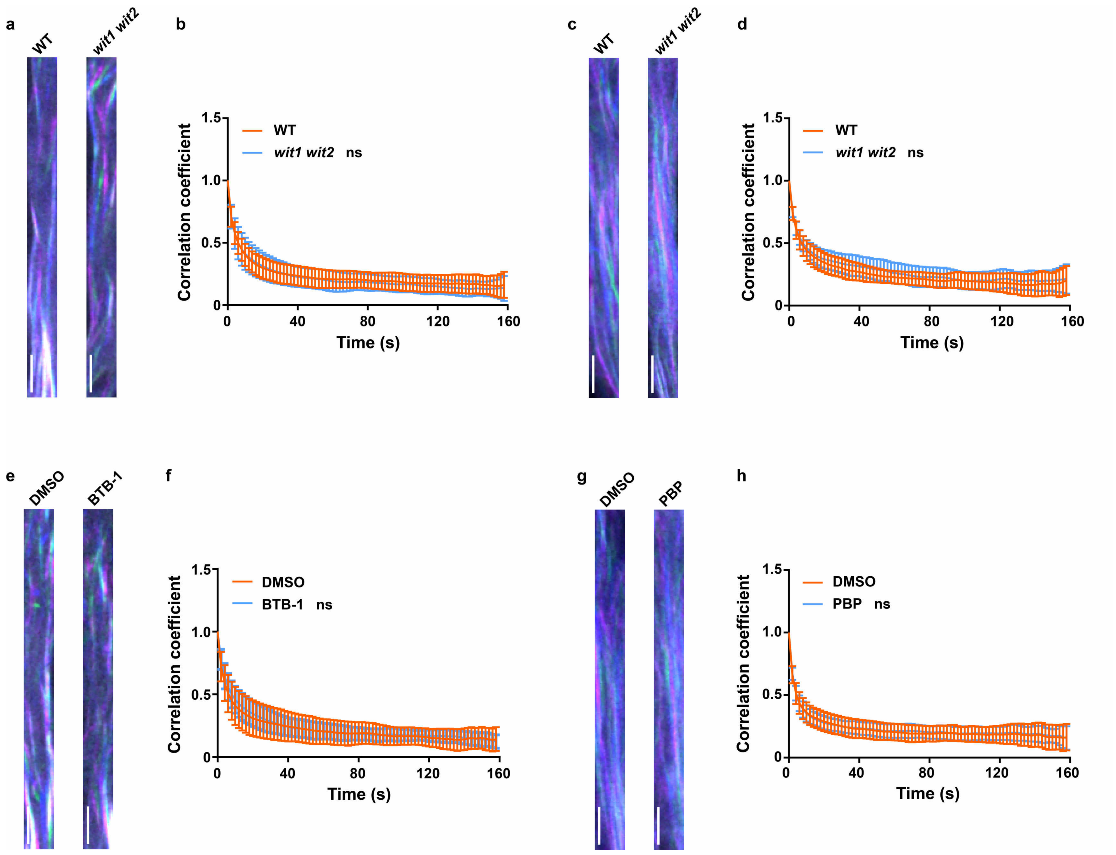

Supplementary Figure 5. Microtubule and actin filament dynamics are not significantly affected in motor proteins inhibitor-treated WT and *wit1 wit2* mutant pollen tubes.

a Representative merged images showing mCherry-MBD-labeled microtubules at 3 time points at 10-s intervals as different colors (magenta, green, and blue) in WT and *wit1 wit2* mutant pollen tubes. Bar, 5 μm. **b** Correlation coefficient was calculated at all possible temporal intervals for time-lapse images of microtubules in WT and *wit1 wit2* mutant pollen tubes. WT (n = 19), *wit1 wit2* (n = 17). **c** Representative merged images showing Lifeact-mEGFP-labeled actin filaments at 3 time points at 10-s intervals as different colors (magenta, green, and blue) in WT and *wit1 wit2* mutant pollen tubes. Bar, 5 μm. **d** Correlation coefficient was calculated at all possible temporal intervals for time-lapse images of actin filaments in WT and *wit1 wit2* mutant pollen tubes. WT (n = 20), *wit1 wit2* (n = 14). **e** Representative merged images showing mCherry-MBD-labeled microtubules at 3 time points at 10-s intervals as different colors (magenta, green, and blue) in DMSO- and BTB-1-treated WT pollen tubes. Bar, 5 μm. **f** Correlation coefficient was calculated at all possible temporal intervals for time-lapse images of microtubules in DMSO- and BTB-1-treated WT pollen tubes. DMSO (n = 12), BTB-1 (n = 14). **g** Representative merged images showing Lifeact-mEGFP-labeled actin filaments at 3 time points at 10-s intervals as different colors (magenta, green, and blue) in DMSO- and PBP-treated WT pollen tubes. Bar, 5 μm. **h** Correlation coefficient was calculated at all possible temporal intervals for time-lapse images of actin filaments in DMSO- and PBP-treated WT pollen tubes. DMSO (n = 17), PBP (n = 22). Values are mean ± SD; ns, not significant; two-tailed Student's *t* test in (**b**), (**d**), (**f**) and (**h**). Source data are provided as a Source Data file.

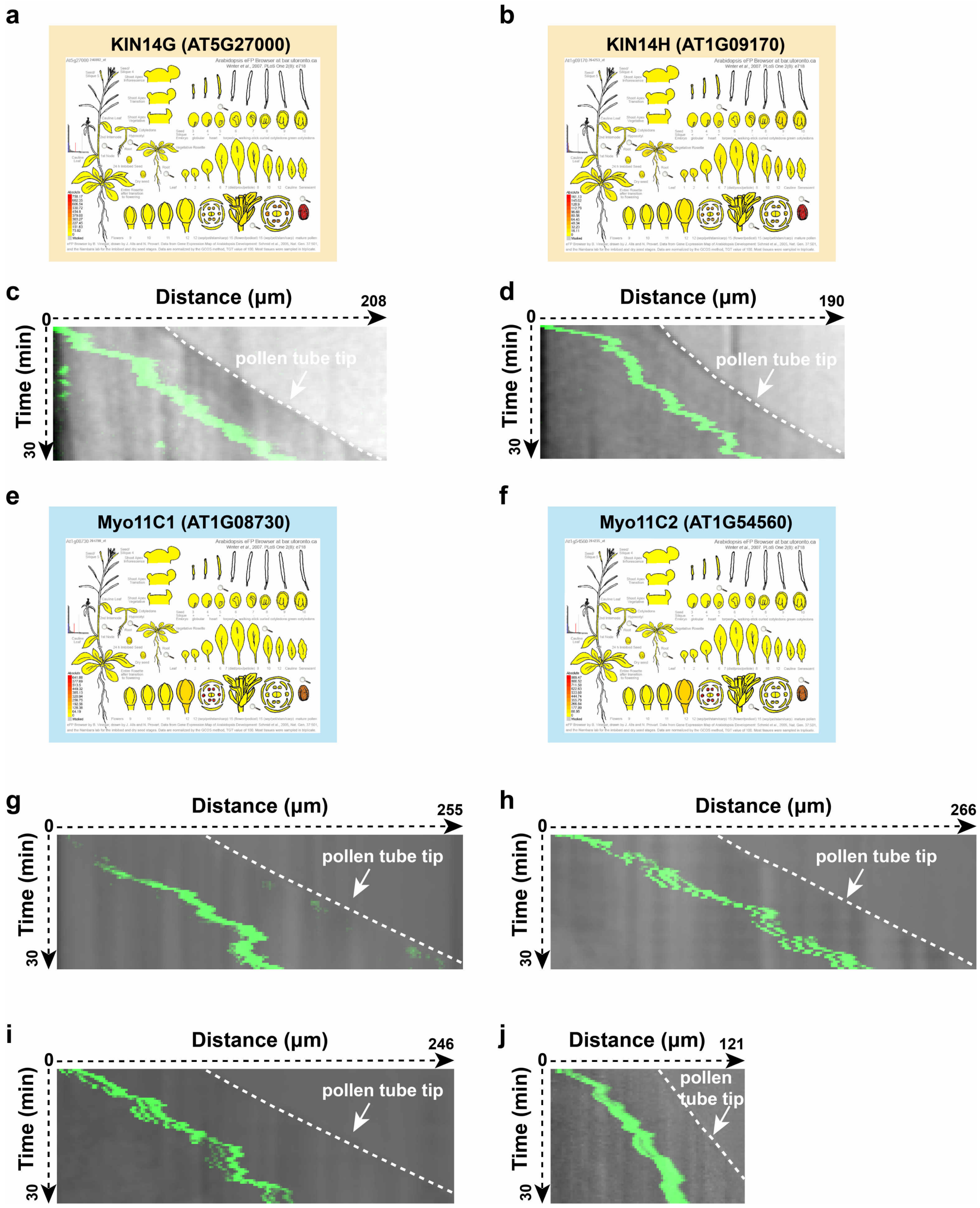

Supplementary Figure 6. Mutations of motor proteins impact on VN movement in pollen tubes.

a, b *KIN14G* and *KIN14H* gene expression in *Arabidopsis thaliana* tissue. (https://bar.utoronto.ca/efp//cgi-bin/efpWeb.cgi). **c, d** Kymograph analysis of the signals of VN in *kin14g* (**c**) and *kin14h* (**d**) mutant. The pollen tube tip is indicated with an arrow. **e, f** *Myo11C1* and *Myo11C2* gene expression in *Arabidopsis thaliana* tissue. (https://bar.utoronto.ca/efp//cgi-bin/efpWeb.cgi). **g-j** Kymograph analysis of the signals of VN and SCs in wild type (**g**), *myo11c1* (**h**), *myo11c2* (**i**) and *myo11c1 myo11c2* double mutant (**j**), respectively. The pollen tube tips are indicated with arrows.

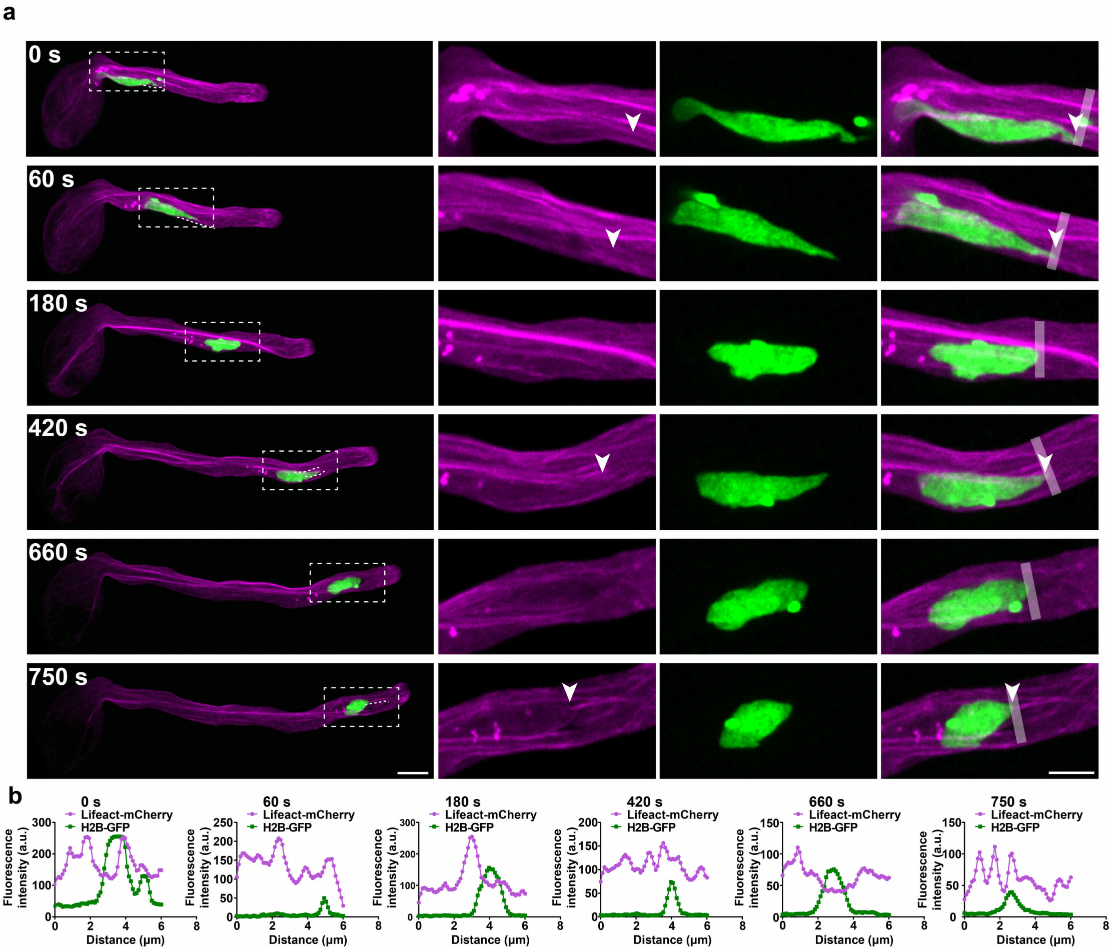

Supplementary Figure 7. The leading edge of VN was only occasionally co-localized with actin filaments.

a Time-lapse imaging showing the dynamics of Lifeact-mCherry and H2B-GFP in fast growing pollen tubes. The region marked with white dotted square is enlarged in the right panels. The leading edge of VN is indicated by arrowheads. Bar in left panel, 10 μm; bar in right panel, 5 μm. **b** Fluorescence intensity of Lifeact-mCherry and H2B-GFP is perpendicular to the leading edge of the VN in (**a**).

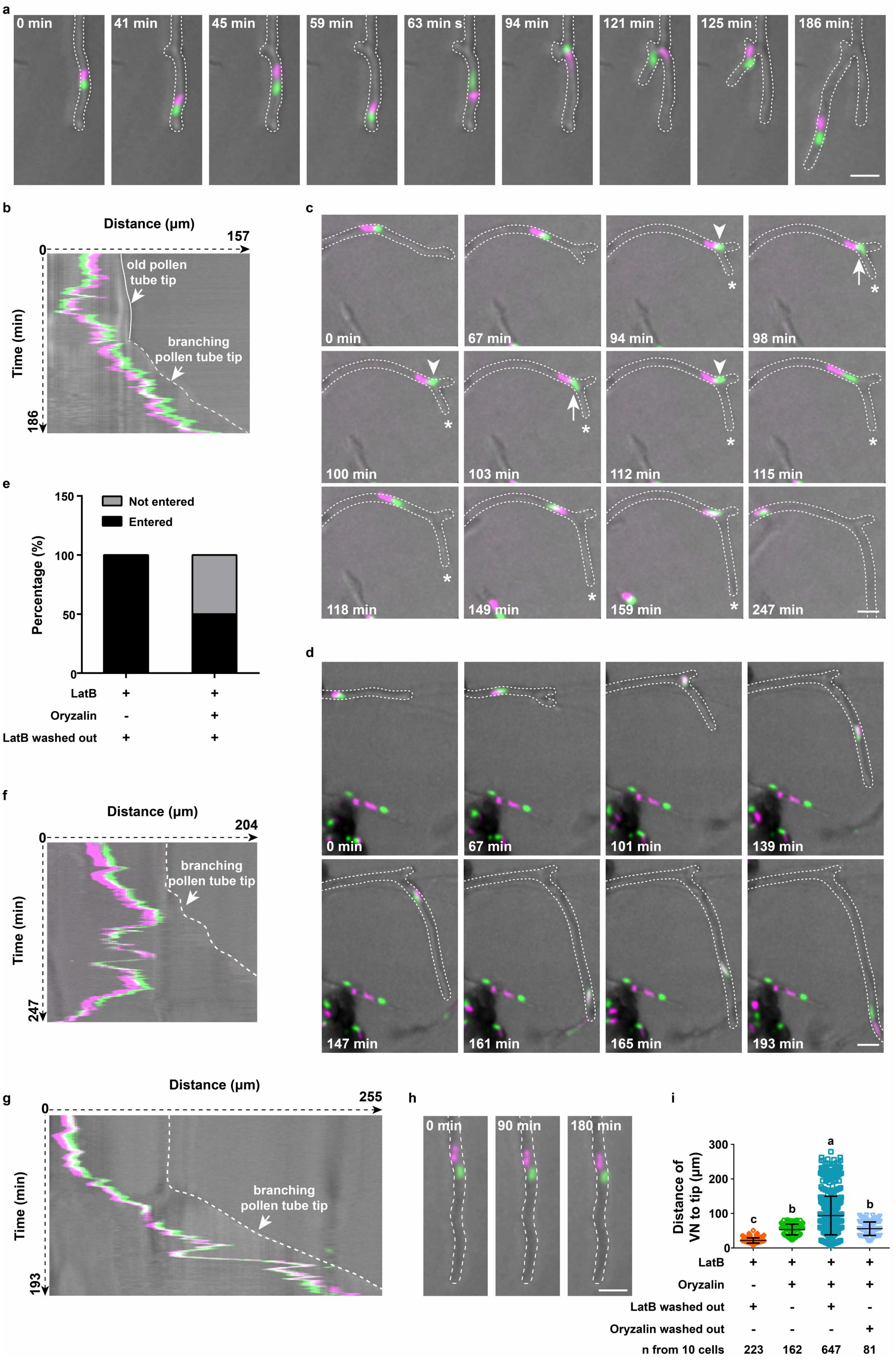

Supplementary Figure 8. Microtubules coordinate the directional movement of MGU.

a Time-lapse images of VN (green) and SCs (magenta) in pollen tubes upon washout of

LatB after LatB treatment. ‘‘0 min’’, the timepoint when pollen was transferred to medium without LatB. Bar, 20 µm. **b** Kymograph analysis of results shown in (**a**). Arrows indicate the old and new pollen tube tips. **c** Time-lapse images of VN and SCs that fail to enter the branched tube tip in pollen tubes treated with the combination of LatB and oryzalin and then transferred to media with oryzalin alone. The new tube tip is marked with an asterisk. The tip of the VN at the branch site of pollen tube is indicated with arrows and arrowheads. ‘‘0 min’’, the timepoint when pollen was transferred to medium with oryzalin alone. Bar, 20 µm. **d** Time-lapse images of VN and SCs that successfully enter the new tube tip in pollen tubes treated with the combination of LatB and oryzalin and then transferred to media with oryzalin alone. ‘‘0 min’’, the timepoint when pollen was transferred to medium with oryzalin but LatB. Bar, 20 µm. **e** The percentage of MGU that enter or fail to enter the new pollen tube in control (LatB washout after LatB treatment) sample and pollen tubes treated with LatB and oryzalin and then transferred to media with oryzalin. n = 20 pollen tubes for each treatment. **f, g** Kymograph analysis of (**c**) and (**d**). **h** Time-lapse images of VN and SCs in pollen tubes upon washout of oryzalin after treatment with the combination of LatB and oryzalin. ‘‘0 min’’, the timepoint when pollen was transferred to medium with LatB alone. Bar, 20 µm. **i** Quantification of the distance between VN and the new pollen tube tip in pollen tubes under LatB washout after LatB treatment, LatB and oryzalin co-treatment, LatB washout after LatB and oryzalin treatment, and oryzalin washout after LatB and oryzalin treatment. Significance was determined by one-way ANOVA analysis. Source data are provided as a Source Data file.

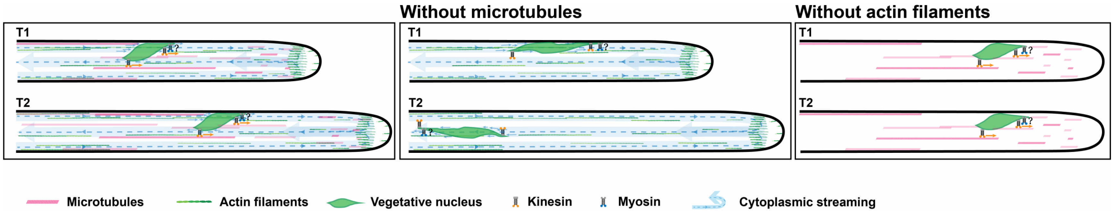

Supplementary Figure 9. The proposed working model of the dynamic interplay among cytoplasmic streaming, the microtubule-kinesin system, and VN in pollen tubes.

In the rapidly growing pollen tubes, the actomyosin-dependent cytoplasmic streaming has significant impact on the velocity and direction of local VN movement, whereas the microtubule-kinesin system finely controls the speed and positioning of VN, allowing the VN to follow and keep pace with the growth of pollen tubes (left panel). Depolymerization of microtubules has minor effect on the cytoplasmic streaming or pollen tube growth. However, the VN loses its stable position to the pollen tube tip and moves in an irregular pattern in pollen tubes in the absence of microtubules (middle panel). When actin filaments are depolymerized, the cytoplasmic streaming, pollen tube growth and VN movement are inhibited (right panel). The blue arrows around the VN indicates the direction of the cytoplasmic circulation encountered by VN. The yellow arrows indicate that the net direction of VN movement toward pollen tube tip is controlled by kinesin. The question marks indicate the possibility that myosin might locate at VN surface and directly participate in VN migration in pollen tubes.

Supplementary Table 1 Generation of fluorescently labeled reporter lines for visualizing microtubules in pollen

Marker line	Pollen grain	Pollen tube
pTUA1:TUA1-GFP/tua1	VN and SCs	VN and SCs
pTUB1:TUB1-GFP/tub1	cytoplasm	cytoplasm
pTUB4:TUB4-GFP/tub4	VN and around SCs	VN and around SCs
pTUB9:TUB9-GFP/tub9	VN and SCs	VN and SCs
pTUA1:mScarlet-TUA1/tua1	cytoplasm	cytoplasm
pTUB1:mScarlet-TUB1/tub1	around SCs	around SCs
pTUB4:mScarlet-TUB4/tub4	cytoplasm	MTs
pTUB9:mScarlet-TUB9/tub9	cytoplasm	MTs
Lat52:mCherry-EB1a/Col-0	cytoplasm	cytoplasm
Lat52:mCherry-EB1b/Col-0	cytoplasm and around VN	cytoplasm and around VN
Lat52:mCherry-EB1c/Col-0	VN	VN
Lat52:mCherry-MBD/Col-0	MTs	MTs

Supplementary Table 2. Primers used in this study.

Name of specific primers		Pairs of specific primers
pTUA1:TUA1-GFP	pTUA1-TUA1	F-ACAGCTATGACCATGATTACGAATTCTCAACCAACTTCCATAGATGCCTAAAATC
		R-CCGCCGCCGCCGGATCCCCGGGTACCATACTCATCGCCTTCTTCGTCG
pTUB1:TUB1-GFP	pTUB1-TUB1	F-ACAGCTATGACCATGATTACGAATTCAGCTAAATGACACGATAATCCC
		R-CCGCCGCCGCCGGATCCCCGGGTACCAGATTCGTAAACTTGTTCTTCTTC
pTUB4:TUB4-GFP	pTUB4-TUB4	F-ACAGCTATGACCATGATTACGAATTCGAGGTAGAAAGGCACAGCATAG
		R-GCCGCCGGATCCCCGGGTACCGAGCTCAGTCTCGTACTCCTCTTCTTCC
pTUB9:TUB9-GFP	pTUB9-TUB9	F-ATGACCATGATTACGAATTCGAGCTCAAAACACACCCTCCTCCTCCTC
		R-ACCCATGCCGCCGCCGCCGCCGGATCCGGCTTCTTCTTCTTCTTCGTCC
pTUA1:mScarlet-TUA1	pTUA1	F-ACGACGGCCAGTGCCAAGCTTCATCTTTGACCTCAACCAAC
		R-CCCTTGCTCACCATGTTCGAAAATTTCTCAGAGACTTC
	mScarlet	F-TTTCGAACATGGTGAGCAAGGGCGAGG
		R-TCTCCCTCATGTCGACGCCGCCGCCGCCGCCCTTGTACAGCTCGTCCATGCC
	TUA1	F-GCGGCGTCGACATGAGGGAGATCATTAGCATTC
		R-CTATGACATGATTACGAATTCCTAATACTCATCGCCTTCTTCG
pTUB1:mScarlet-TUB1	pTUB1	F-ACGACGGCCAGTGCCAAGCTTAGCTAAATGACACGATAATCCC
		R-CCCTTGCTCACCATGATGATGATGATCGATGAAGATTTTG
	mScarlet	F-CATCATCATGGTGAGCAAGGGCGAGG
		R-TTTCTCTCATGTCGACGCCGCCGCCGCCGCCCTTGTACAGCTCGTCCATGCC

	TUB1	F-GCGGCGTCGACATGAGAGAAATCCTCCACGTC
		R-CTATGACATGATTACGAATTCTCAAGATTCGTAAACTTGTTCTTCTTC
pTUB4:mScarlet-TUB4	pTUB4	F-ACGACGGCCAGTGCCAAGCTTGAGGTAGAAAGGCACAGCATAG
		R-CCCTTGCTCACCATTTTTTTTTTTTTTGGTTTCTTCGTCAAGAGC
	mScarlet	F-AAAAAAAATGGTGAGCAAGGGCGAGGC
		R-TCTCTCTCATGTCGACGCCGCCGCCGCCCTTGTACAGCTCGTCCATGCC
	TUB4	F-TCTCTCTCATGTCGACGCCGCCGCCGCCCTTGTACAGCTCGTCCATGCC
		R-CTATGACATGATTACGAATTCTTAAGTCTCGTACTCCTCTTCTTCCTC
pTUB9:mScarlet-TUB9	pTUB9	F-ACGACGGCCAGTGCCAAGCTTAAAACACACCCTCCTCCTCCTC
		R-CCCTTGCTCACCATTTTCACTCGGATGTTTTTTTTTTGTTC
	mScarlet	F-GAGTGAAAATGGTGAGCAAGGGCGAGGC
		R-TTTCTCTCATGTCGACGCCGCCGCCGCCCTTGTACAGCTCGTCCATGCC
	TUB9	F-GCGGCGTCGACATGAGAGAAATTCTTCATATTCAAGGAGG
		R-CTATGACATGATTACGAATTCTTAGGCTTCTTCTTCTTCTTCGTCC
Lat52:mCherry-Eb1a	Lat52	F-GATACACCAAATCGACTCTAGAGTCGACATACTCGACTCAGAAGGT
		R-TCGCCCTTGCTCACCATGGTACCTTTAAATTGGAATTTTTTTTTTTGGTGTGTGT
	mCherry	F-CCAATTTAAAGGTACCATGGTGAGCAAGGGCG
		R-TCGCCAGCCGCCGCCGCCGCCCTTGTACAGCTCGTCCATGC
	EB1a	F-GGCGGCGGCGGCGGCTGGCGACGAACATCGGAATGA
		R-CGATCGGGGAAATTCGAGCTCTTAGGCTTGAGTCTTTTCTTC

Lat52:mCherry-Eb1b	Lat52	F-GATACACCAAATCGACTCTAGAGTCGACATACTCGACTCAGAAGGT
		R-TCGCCCTTGCTCACCATGGTACCTTTAAATTGGAATTTTTTTTTTTGGTGTGTGT
	mCherry	F-CCAATTTAAAGGTACCATGGTGAGCAAGGGCG
		R-GTCGCCATGCCGCCGCCGCCGCCCTTGTACAGCTCGTCCATGC
	EB1b	F-GGCGGCGGCGGCGGCATGGCGACGAACATTGGGATG
		R-CGATCGGGGAAATTCGAGCTCTTAAGTTTGGGTCTCTGCAGCAG
Lat52:mCherry-Eb1c	Lat52	F-GATACACCAAATCGACTCTAGAGTCGACATACTCGACTCAGAAGGT
		R-TCGCCCTTGCTCACCATGGTACCTTTAAATTGGAATTTTTTTTTTTGGTGTGTGT
	mCherry	F-CCAATTTAAAGGTACCATGGTGAGCAAGGGCG
		R-TAGCCATGCCGCCGCCGCCGCCCTTGTACAGCTCGTCCATGC
	EB1c	F-GGCGGCGGCGGCGGCATGGCTACGAACATTGGGATG
		R-CGATCGGGGAAATTCGAGCTCTCAGCAGGTCAAGAGAGGAGATG
Lat52:mCherry-MBD	Lat52	F-GATACACCAAATCGACTCTAGAGTCGACATACTCGACTCAGAAGGT
		R-TCGCCCTTGCTCACCATGGTACCTTTAAATTGGAATTTTTTTTTTTGGTGTGTGT
	mCherry	F-CCAATTTAAAGGTACCATGGTGAGCAAGGGCG
		R-GACATGCCGCCGCCGCCGCCCTTGTACAGCTCGTCCATGC
	MBD	F-GGCGGCGGCGGCGGCATGTCCCGGCAAGAAGAAGC
		R-CGATCGGGGAAATTCGAGCTCTTAACCTCCTGCAGGAAAGTGG
Lat52:mEGFP-MBD	Lat52	F-GATACACCAAATCGACTCTAGAGTCGACATACTCGACTCAGAAGGT
		R-TCGCCCTTGCTCACCATGGTACCTTTAAATTGGAATTTTTTTTTTTGGTGTGTGT

mEGFP	F-AAATTCCAATTTAAAGGTACCATGGTGAGCAAGGGCGAG
	R-GGACATGCCGCCGCCGCCGCCGCCCTTGTACAGCTCGTCCATGC
MBD	F-GGCGGCGGCGGCGGCGGCATGTCCCGGCAAGAAGAAGC
	R-CGATCGGGGAAATTCGAGCTCTTAACCTCCTGCAGGAAAGTGG
tua1 (SALK_097254)	F-GAGATATGTGGAAGGGGATGAG
	R-GTTGAATGCATCATGACATGC
tub1 (SALK_036755)	F-TGTGTTGACACTTTCGAGGTG
	R-GCTTCAGTGAACTCCATCTCG
tub4 (SALK_204506)	F-GGTTCGTCACATTGGTCAAAG
	R-AAAACGCTGACGAGTGTATGG
tub9 (SALK_015876C)	F-TTTTTGTTTGAACCGTTGATTC
	R-AGCAGATCTGAATTCGAATGC
kin14g (SALK_133582)	F-CGGGTCAGGGAAAACTTTTAC
	R-CTCCGAGAGTGTCAGGTTCTG
kin14h (SALK_106474)	F-GTTCTCAGGGAGGTAAATCCG
	R-TTGTTGCTTCTCGGTCTTCTC
myo11c1 (SALK_129231C)	F-GGAGCTGGAAAAACTGAAACC
	R-TTGATAACTTCCTCGGGTGTG
myo11c2 (SALK_089338C)	F-GCAGAGGTGAACCATCTGATTC
	R-ATCTGATCGCCAATGAATACG

wit1 (GABI_470E06)	F-TTTGATCTTAATCGTCGTGTCG
	R-AGATCACCAAACTCACCATCG
wit2 (SALK_127765)	F-TGCCTCTATTTCGTGACCATC
	R-ATCTTCTCGGATGGAAGAAGC
LBb1.3	ATTTTGCCGATTTCGGAAC
GABI	ATAATAACGCTGCGGACATCTACATTTT
