# Supplementary material for: Distinct functions of microtubules and actin filaments in the transportation of the male germ unit in pollen
Source: Nat Commun. 2024 Jun 27;15:5448. doi: 10.1038/s41467-024-49323-9 (PMC11211427; doi:10.1038/s41467-024-49323-9)
Supplement: Supplementary file 3 — Description of additional supplementary files [file 41467_2024_49323_MOESM3_ESM.docx]

**Description of Additional Supplementary Files:**

**Supplementary Movie 1:** Dynamics of microtubules and actin filaments in a germinating pollen grain (corresponding to Figure 2d). Bar = 5 µm.

**Supplementary Movie 2:** Dynamics of microtubules and VN in germinating pollen grains without (upper pannels) or with (lower pannels) oryzalin treatment (corresponding to Figure 3c, 4a). Bar = 5 µm.

**Supplementary Movie 3:** Dynamics of microtubules and VN in a growing pollen tube (corresponding to Figure 3e). The contact sites of microtubules and the leading edge of VN are indicated with arrowheads. The contact sites of microtubules and the trailing edge of VN are indicated with arrows. Bar = 10 µm.

**Supplementary Movie 4:** Dynamics of microtubules and VN in a pollen tube after oryzalin washout (corresponding to Figure 4k). A high dose of oryzalin was applied to completely depolymerize microtubules in pollen tubes before transferring the pollen tubes to inhibitor-free media. “0 s” was set at the timepoint when pollen grains were transferred to medium without oryzalin. The reassembly sites of microtubules are indicated by arrows, and the contact sites of VN tips and microtubules are pointed out with arrowheads. Bar = 10 µm.

**Supplementary Movie 5:** Dynamics of microtubules and VN in a pollen tube after oryzalin and BTB-1 co-treatment and followed by oryzalin washout (corresponding to Figure 5m). Oryzalin and BTB-1 were simultaneously applied to pollen tubes, before transferring the pollen tubes to media supplemented with BTB-1 alone. ‘‘0 s’’ was set at the timepoint when pollen was transferred to medium with BTB-1 alone. The reassembly site of microtubules is indicated by arrows, and the contact sites of VN tips and microtubules are pointed out with arrowheads. Bar, 10 μm.

**Supplementary Movie 6:** Cytoplasmic streaming impacts on VN migration in pollen tubes (corresponding to Figure 6a, b, c). Forward movement of VN in upper panel, pause of VN migration in middle panel, backward movement of VN in lower panel. The black arrows in pollen tube indicates the vector distribution of the velocity of vesicles. The arrowheads indicate the tube growth direction. Bars, 10 μm.

**Supplementary Movie 7:** Dynamics of actin filaments and VN in a germinating pollen grain (corresponding to Figure 6e). Bar = 5 µm.

**Supplementary Movie 8:** Dynamics of actin filaments and VN in a growing pollen tube (corresponding to Supplementary Figure 7a). The contact sites of actin filaments and the leading edge of VN are indicated with arrowheads. The contact sites of actin filaments and the trailing edge of VN are indicated with arrows. Bar = 10 µm.
