# Supplementary material for: Distinct functions of microtubules and actin filaments in the transportation of the male germ unit in pollen
Source: Nat Commun. 2024 Jun 27;15:5448. doi: 10.1038/s41467-024-49323-9 (PMC11211427; doi:10.1038/s41467-024-49323-9)
Supplement: Supplementary file 12 — Reporting summary [file 41467_2024_49323_MOESM12_ESM.pdf]

nature portfolio

Corresponding author(s): Haiyun Ren

Last updated by author(s): May 12, 2024

Reporting Summary

Nature Portfolio wishes to improve the reproducibility of the work that we publish. This form provides structure for consistency and transparency in reporting. For further information on Nature Portfolio policies, see our Editorial Policies and the Editorial Policy Checklist.

Statistics

For all statistical analyses, confirm that the following items are present in the figure legend, table legend, main text, or Methods section.

n/a	Confirmed	
☐	☒	The exact sample size (*n*) for each experimental group/condition, given as a discrete number and unit of measurement
☐	☒	A statement on whether measurements were taken from distinct samples or whether the same sample was measured repeatedly
☐	☒	The statistical test(s) used AND whether they are one- or two-sided<br>*Only common tests should be described solely by name; describe more complex techniques in the Methods section.*
☐	☒	A description of all covariates tested
☐	☒	A description of any assumptions or corrections, such as tests of normality and adjustment for multiple comparisons
☐	☒	A full description of the statistical parameters including central tendency (e.g. means) or other basic estimates (e.g. regression coefficient) AND variation (e.g. standard deviation) or associated estimates of uncertainty (e.g. confidence intervals)
☐	☒	For null hypothesis testing, the test statistic (e.g. *F*, *t*, *r*) with confidence intervals, effect sizes, degrees of freedom and *P* value noted<br>*Give P values as exact values whenever suitable.*
☒	☐	For Bayesian analysis, information on the choice of priors and Markov chain Monte Carlo settings
☒	☐	For hierarchical and complex designs, identification of the appropriate level for tests and full reporting of outcomes
☐	☒	Estimates of effect sizes (e.g. Cohen's *d*, Pearson's *r*), indicating how they were calculated

Our web collection on statistics for biologists contains articles on many of the points above.

Software and code

Policy information about availability of computer code

Data collection	Imaging data was collected using Volocity software accompanied with a spinning disk confocal microscope (UltraView VoX, PerkinElmer, UK), Zeiss ZEN Blue software accompanied with confocal microscope (LSM 710/880, Zeiss, DE), Fusion software accompanied with a high-speed laser confocal living cell workstation (Dragonfly, Andor, UK) in this study.
Data analysis	The acquired images were processed using Imaris (Bitplane), Zen Blue software (Zeiss) , ImageJ (https:// imagej.nih.gov/ij/) and PIVlab (http://ww2.mathworks.cn/matlabcentral/fileexchange/27659-pivlab-particle-image-velocimetry-piv-tool-with-gui?s_tid=ta_fx_results). Microsoft Excel and Graphpad Prism were used for statistical analysis.

For manuscripts utilizing custom algorithms or software that are central to the research but not yet described in published literature, software must be made available to editors and reviewers. We strongly encourage code deposition in a community repository (e.g. GitHub). See the Nature Portfolio guidelines for submitting code & software for further information.

Data

Policy information about availability of data

All manuscripts must include a data availability statement. This statement should provide the following information, where applicable:

- Accession codes, unique identifiers, or web links for publicly available datasets
- A description of any restrictions on data availability
- For clinical datasets or third party data, please ensure that the statement adheres to our policy

All data supporting the findings of this study are available in the Source Data or the Supplementary materials.

Research involving human participants, their data, or biological material

Policy information about studies with human participants or human data. See also policy information about sex, gender (identity/presentation), and sexual orientation and race, ethnicity and racism.

Reporting on sex and gender	Not applicable.
Reporting on race, ethnicity, or other socially relevant groupings	Not applicable.
Population characteristics	Not applicable.
Recruitment	Not applicable.
Ethics oversight	Not applicable.

Note that full information on the approval of the study protocol must also be provided in the manuscript.

Field-specific reporting

Please select the one below that is the best fit for your research. If you are not sure, read the appropriate sections before making your selection.

☒ Life sciences ☐ Behavioural & social sciences ☐ Ecological, evolutionary & environmental sciences

For a reference copy of the document with all sections, see nature.com/documents/nr-reporting-summary-flat.pdf

Life sciences study design

All studies must disclose on these points even when the disclosure is negative.

Sample size	No statistical methods were used to predetermine sample sizes. Sample size was determined based on previous experimental experience with similar assays and/or the sample size generally employed in the field
Data exclusions	No data were excluded from the analysis.
Replication	All experiments were performed multiple times. Exact replication numbers are in the figure legends, the results or the methods.
Randomization	Samples of the same genotypes were randomly collected.
Blinding	The investigators were not blinded to allocation during experiment and assessment because phenotypes is clear.

Reporting for specific materials, systems and methods

We require information from authors about some types of materials, experimental systems and methods used in many studies. Here, indicate whether each material, system or method listed is relevant to your study. If you are not sure if a list item applies to your research, read the appropriate section before selecting a response.

Materials & experimental systems

n/a	Involved in the study	
☐	☒	Antibodies
☒	☐	Eukaryotic cell lines
☒	☐	Palaeontology and archaeology
☒	☐	Animals and other organisms
☒	☐	Clinical data
☒	☐	Dual use research of concern
☐	☒	Plants

Methods

n/a	Involved in the study	
☒	☐	ChIP-seq
☒	☐	Flow cytometry
☒	☐	MRI-based neuroimaging

Antibodies

Antibodies used	Primary antibody:anti-β tubulin (Abcam, ab15568). Secondary antibody: goat anti-rabbit IgG Alexa Fluor 594 (Abcam, ab150080).
Validation	Anti-β tubulin is rabbit polyclonal to beta Tubulin, and has been validated by others and cited by 85 publications.<br>Goat anti-rabbit IgG Alexa Fluor 594 has been validated by others and cited by 592 publications.

Dual use research of concern

Policy information about dual use research of concern

Hazards

Could the accidental, deliberate or reckless misuse of agents or technologies generated in the work, or the application of information presented in the manuscript, pose a threat to:

No	Yes	
☒	☐	Public health
☒	☐	National security
☒	☐	Crops and/or livestock
☒	☐	Ecosystems
☒	☐	Any other significant area

Experiments of concern

Does the work involve any of these experiments of concern:

No	Yes	
☒	☐	Demonstrate how to render a vaccine ineffective
☒	☐	Confer resistance to therapeutically useful antibiotics or antiviral agents
☒	☐	Enhance the virulence of a pathogen or render a nonpathogen virulent
☒	☐	Increase transmissibility of a pathogen
☒	☐	Alter the host range of a pathogen
☒	☐	Enable evasion of diagnostic/detection modalities
☒	☐	Enable the weaponization of a biological agent or toxin
☒	☐	Any other potentially harmful combination of experiments and agents

Plants

Seed stocks	The T-DNA insertional mutants, tua1 (SALK_097254), tub1 (SALK_036755), tub4 (SALK_204506) and tub9 (SALK_015876C), kin14g (SALK_133582), kin14h (SALK_106474), myo11c1 (SALK_129231C), myo11c2 (SALK_089338C), wit1 (GABI_470E06) and wit2 (SALK_127765) were obtained from NASC (http://arabidopsis.info/).
Novel plant genotypes	Double mutants were obtained by crossing corresponding single mutant. myo11c1 myo11c2 double mutant was kindly shared by Prof. Andreas Nebenfuhr.<br>The transformation of Arabidopsis was performed by floral dip. At least two independent lines of T3 generation plants were analyzed.
Authentication	The transformations were selected by antibiotic resistance and fluorescence intensity.
